# A matrigel-free method for culture of pancreatic endocrine-like cells in defined protein-based hydrogels

**DOI:** 10.3389/fbioe.2023.1144209

**Published:** 2023-03-09

**Authors:** Mark T. Kozlowski, Heather N. Zook, Desnor N. Chigumba, Christopher P. Johnstone, Luis F. Caldera, Hung-Ping Shih, David A. Tirrell, Hsun Teresa Ku

**Affiliations:** ^1^ Division of Chemistry and Chemical Engineering, California Institute of Technology, Pasadena, CA, United States; ^2^ Department of Translational Research and Cellular Therapeutics, Arthur Riggs Diabetes and Metabolism Research Institute and Beckman Research Institute of City of Hope, Duarte, CA, United States; ^3^ The Irell and Manella Graduate School of Biological Sciences, City of Hope, Duarte, CA, United States

**Keywords:** defined culture medium, endocrine cells, matrigel-free culture, pancreatic development, protein-based culture, pancreatic organoid, protein engineered hydrogel, rheology

## Abstract

The transplantation of pancreatic endocrine islet cells from cadaveric donors is a promising treatment for type 1 diabetes (T1D), which is a chronic autoimmune disease that affects approximately nine million people worldwide. However, the demand for donor islets outstrips supply. This problem could be solved by differentiating stem and progenitor cells to islet cells. However, many current culture methods used to coax stem and progenitor cells to differentiate into pancreatic endocrine islet cells require Matrigel, a matrix composed of many extracellular matrix (ECM) proteins secreted from a mouse sarcoma cell line. The undefined nature of Matrigel makes it difficult to determine which factors drive stem and progenitor cell differentiation and maturation. Additionally, it is difficult to control the mechanical properties of Matrigel without altering its chemical composition. To address these shortcomings of Matrigel, we engineered defined recombinant proteins roughly 41 kDa in size, which contain cell-binding ECM peptides derived from fibronectin (ELYAVTGRGDSPASSAPIA) or laminin alpha 3 (PPFLMLLKGSTR). The engineered proteins form hydrogels through association of terminal leucine zipper domains derived from rat cartilage oligomeric matrix protein. The zipper domains flank elastin-like polypeptides whose lower critical solution temperature (LCST) behavior enables protein purification through thermal cycling. Rheological measurements show that a 2% w/v gel of the engineered proteins display material behavior comparable to a Matrigel/methylcellulose-based culture system previously reported by our group to support the growth of pancreatic ductal progenitor cells. We tested whether our protein hydrogels in 3D culture could derive endocrine and endocrine progenitor cells from dissociated pancreatic cells of young (1-week-old) mice. We found that both protein hydrogels favored growth of endocrine and endocrine progenitor cells, in contrast to Matrigel-based culture. Because the protein hydrogels described here can be further tuned with respect to mechanical and chemical properties, they provide new tools for mechanistic study of endocrine cell differentiation and maturation.

## Introduction

Type 1 diabetes (T1D) is an autoimmune disorder that results in the destruction of a patient’s pancreatic endocrine beta cells, which prevents insulin secretion and endogenous regulation of blood glucose levels. This condition affects approximately nine million people worldwide according to the World Health Organization (Xu et al., 2018), and can lead to serious complications such as coronary artery disease, retinopathy, and diabetic nephropathy resulting in kidney failure (Bjornstad et al., 2015). Currently, T1D is treated by a combination of injections of exogenous insulin and careful regulation of diet and lifestyle. Although life-saving, insulin injection does not completely prevent glucose excursion or the associated long-term complications (Nalysnyk et al., 2010). A promising alternative therapy is the transplantation of cadaveric human islets, which has been used effectively in over 1,500 patients worldwide since the first report of a successful allogenic transplant in the year 2000 (Shapiro et al., 2000; Shapiro et al., 2017). However, clinical demands outpace transplant supplies, as multiple donors are typically required for each recipient (Walker et al., 2022), and repeated transplantations into a single recipient over time may be required due to progressive graft failure caused by allo- and auto-immunity (Moore et al., 2015).

To meet the demand for transplantable beta cells, several groups have developed methods for the production of functional beta-like cells from pluripotent stem cells capable of treating insulin-dependent diabetic mice (Pagliuca et al., 2014; Rezania et al., 2014; Ma et al., 2018a; Ma et al., 2018b; Sui et al., 2018; Nair et al., 2019; Maxwell et al., 2020; Hogrebe et al., 2021; Du et al., 2022; Hebrok et al., 2022; Maxwell et al., 2022; Parent et al., 2022), and clinical trials of similar techniques are underway in humans (de Klerk and Hebrok, 2021; Migliorini et al., 2021; Butler and Gale, 2022). However, in order to produce transplantable cells, progenitor cells require appropriate biochemical and physical cues to promote beta cell formation, a process that is still not fully elucidated. Currently, many methods rely on the use of Matrigel, a secretion of Engelbreth-Holm-Swarm mouse sarcoma cells enriched for extracellular matrix (ECM) proteins. Matrigel is undefined and extremely complex, with one proteomic profile showing over 1,800 unique proteins (Hughes et al., 2010). The undefined nature of Matrigel makes it difficult to determine exactly which factors are responsible for the differentiation of pluripotent stem cells towards a particular lineage. Matrigel also suffers from lot-to-lot variation, which affects cellular fate decisions (Vukicevic et al., 1992; Goldstein et al., 2011). Furthermore, the xenogeneic origin of Matrigel impedes its use in clinical applications (Ludwig et al., 2006; Unger et al., 2008). Transplanted human pluripotent stem cells mixed with Matrigel potentiate the formation of cancer cells, such as teratomas, in mice (Hentze et al., 2009; Prokhorova et al., 2009; Vaillant et al., 2011). The mechanical properties of Matrigel are also difficult to tune independently of its chemical properties, which is a critical shortcoming because the mechanical environment surrounding the developing stem cells can also affect their differentiation (Engler et al., 2006). Another major challenge for stem cell transplantation in T1D is the immaturity of the *in-vitro* differentiated beta-like cells (Melton, 2021). This is partly due to a lack of suitable assay system that allows the examination of signals important for differentiation of primary, non-stem cell derived, endocrine progenitor cells (Engler et al., 2006; Mamidi et al., 2018).

Both fibronectin (Nakamura et al., 2022) and laminin (Tjin et al., 2014) are known to be important in pancreatic endocrine functions. For these reasons, in this study we engineered two novel recombinant proteins, which we call PEP-FN or PEP-LAMA3, that are well-defined, gel-forming, and equipped with cell-binding motifs. PEP-FN uses an arginine-glycine-aspartic acid (RGD) integrin-binding domain derived from fibronectin (Ruoslahti and Pierschbacher, 1987), and the PEP-LAMA3 protein uses a laminin alpha-3 cell-binding domain previously reported by Tjin and co-workers (Tjin et al., 2014). Previous work characterized recombinant triblock protein, which we called PEP (Dooling and Tirrell, 2016; Rapp et al., 2017; Rapp et al., 2018), a synthetic domain with coiled-coil helix domains that self-associate and give the gel its material properties. These proteins were produced in quantities of hundreds of milligrams in *Escherichia coli* under conventional culture conditions. We tested the ability of the PEP-FN and PEP-LAMA3 proteins to support the growth of primary endocrine and endocrine progenitor cells isolated from young (8-day-old) mice in 3-dimensional (3D) space. Prior studies demonstrated that pancreatic progenitor cells are present in these young mice and are suitable for *in vitro* modeling of cell differentiation to endocrine lineages (which include beta cells among others) (Ghazalli et al., 2015). Here, we demonstrate that the PEP-FN and PEP-LAMA3 proteins support endocrine lineage and endocrine progenitor cells, compared to the control culture containing Matrigel that favors ductal cell formation (Ghazalli et al., 2015).

## Materials and methods

*Protein Expression and Purification*. DNA sequences that encode for PEP-FN or PEP-LAMA3 proteins (Supplementary Table S1) were constructed in the pQE80-L plasmid (Qiagen, Hilden, Germany) and expressed in *E. coli* strain BL21 DE3 in Terrific Broth (TB). From N-terminal to C-terminal, the proteins contain a 6x-histidine tag, a rat cartilage oligomeric matrix protein domain (coiled-coil P) with mutation I58A, an elastin-like polypeptide domain (E), a fibronectin-derived RGD cell-binding domain or a laminin alpha three mimetic, another E domain, and another P domain with mutation I58A. The 6x-histidine tag enabled purification using a Ni-NTA column. Additional details, including steps taken to remove bacterial endotoxin, are presented in supporting information. Both proteins were obtained in yields of ∼100 mg/L of bacterial culture after purification.

*Rheology.* Rheological studies were conducted on a TA Scientific ARES rheometer with TA Orchestrator software. A plate-and-cone geometry with a diameter of 25 mm, cone angle of 0.0396 radians, and a gap of 0.0483 mm (TA Scientific, New Castle, DE) were used. The sample was warmed to 37°C by a Peltier plate. A thin ring of paraffin oil (JT Baker, VWR, Radnor, PA) was placed around the sample to prevent evaporation. A strain sweep was first conducted to establish the linear viscoelastic region of the material, at a frequency of 10 rad/s. Based on these results, frequency sweeps were conducted at a maximum strain of 1%.

*Mice and pancreatic cell preparation*. Animal experiments were conducted under the supervision of the Institutional Animal Care and Use Committee at City of Hope. Adult C57BL/6J (B6) mice (The Jackson Laboratory, Bar Harbor, ME) were purchased and bred at City of Hope. Pancreases were procured from pooled 8-day-old mice (*n* = 5–10) of both sexes, placed into Petri dishes kept on ice which contained approximately 20 ml Phosphate Buffered Saline (PBS) solution supplemented with 0.1% bovine serum albumin (BSA, Sigma-Aldrich, Milwaukee, WI) and 100 units/ml penicillin and 100 μg/ml streptomycin (PS) (Gibco, Grand Island, NY). The spleen and fat surrounding the pancreas were removed with tweezers under a dissecting microscope. The pancreases were washed twice in fresh PBS, and thoroughly minced using spring scissors. Cold PBS/BSA/PS was added, supplemented by 2 units of bovine pancreatic DNase I (EMD Millipore, Temecula, CA) and 2 mg Collagenase B (Sigma-Aldrich) per mL. The pancreases were then pipetted up and down several times to disrupt the tissue, and incubated in a water bath at 37°C for 15 min with additional pipetting every 5 min. The cell suspension was placed in 25 mL cold PBS/BSA/PS/DNase I, and centrifuged at 800 *g* for 5 min. The supernatant was removed, and the cells were resuspended in 1 ml of PBS/BSA/PS/DNase I. The cell suspension was filtered sequentially through a 100 and 40 μm mesh filter, and once again centrifuged. The cells were finally resuspended in approximately 500 μl of PBS/BSA/PS/DNase I, stained with 0.02% Trypan Blue, and manually counted on a hemacytometer.

*Cell plating*. The culture medium consisted of either 5% (v/v) Matrigel (Corning, Manassas, VA) + 1% (w/v) methylcellulose (Wedeken et al., 2017) or a 2% (w/v) solution of proteins PEP-FN or PEP-LAMA3. The liquid medium consisted of Dulbecco’s Modified Eagle Medium/Nutrient Mixture F-12 (DMEM/F12) containing L-glutamine and 15 mM HEPES (Corning), 10% KnockOut Serum Replacement (KSR, ThermoFisher Scientific), 10 mM nicotinamide, and 25 ng/ml recombinant human epidermal growth factor (EGF). PEP-FN or PEP-LAMA3 was dissolved in DMEM/F12 at slightly above working concentration (approximately 4%–5%), and allowed to dissolve overnight at 4°C prior to use. Dissociated pancreatic cells were added at a concentration of 10,000 cells (for PEP-FN and PEP-LAMA3) or 2,500 cells (for Matrigel-methylcellulose, MM) per 100 uL of medium per well in flat-bottomed 96-well uncoated plates (ThermoFisher, Waltham, MA). Sterile distilled water (150 μl) was added to the wells at the edge of the plate to prevent evaporation. Control MM conditions consisted of a 1% (w/v) methylcellulose and 1% (v/v) Matrigel medium in DMEM/F-12 with the same 2-factor growth medium. Cells were plated in triplicate wells, unless specified otherwise, and incubated at 37°C in a 5% CO_2_ atmosphere in a stationary, humidified, mammalian cell incubator.

*Colony counting and analyses*. Pancreatic colonies were observed under an Olympus CKX31 phase-contrast microscope with a ×10 objective lens and counted using a manual differential counter. All colonies grown in Matrigel-methylcellulose media were analyzed on day 4 post-plating due to their rapid growth, whereas those grown in PEP-FN and PEP-LAMA3 media were analyzed on day 7.

*Whole-mount immunostaining*. Pancreatic cell samples were fixed overnight at 4°C in 4% paraformaldehyde (PFA) in PBS, by directly adding the PFA into the culture wells. The samples were pooled, centrifuged at 400 *g* for 15 min to pellet, and the supernatant removed. The samples were washed in PBS, with three changes of wash buffer, and left on a rocking table overnight at 4°C. The PBS was removed, replaced with blocking buffer, and incubated overnight. Primary antibodies (Supplementary Table S2) were added and incubated overnight at 4°C, followed by three washes of PBS supplemented by 0.15% Tween-20 (PBST), and a fourth overnight wash in PBST. Donkey secondary antibodies (Supplementary Table S2) were then added and incubated overnight at 4°C, followed by washing for three times, with the fourth wash for 3 days at 4°C in PBST with rocking and daily changes of wash buffer. The samples were stained with DAPI to visualize the nucleus prior to imaging using an AxioObserver Z1 microscope with an ApoTome attachment (Carl Zeiss Inc., Oberkochen, Germany) and a ×20 objective lens, with the averaging of three ApoTome images presented.

*Micromanipulation of a single pancreatic colony and microfluidic qRT-PCR*. Detailed procedures for micromanipulation and microfluidic qRT-PCR were previously described (Tremblay et al., 2016). Colonies of interest in the culture medium were photographed using an Olympus CKX41 optical microscope with a Luminera Infinity2 camera attachment. Individual pancreatic colonies were hand-picked using a pipette tip in a volume <3 uL. The colonies were placed in a mixture consisting of 5.0 μL of 2x reaction mix, 2.5 μl of Taqman probe mix, 0.2 μL of SuperScript III enzyme, and 1.3 μL TE buffer, following manufacturer instructions for the SuperScript III reverse transcription kit (Thermo-Fisher). The colonies were then subjected to amplification in a Veriti 96-well thermal cycler (Applied Biosystems, Foster City, CA) using the following cycles: 15 min at 55°C, then 22 cycles alternating between 95°C for 15 s and 65°C for 4 min, before being lowered to a temperature of 4°C. The pre-amplified samples were frozen at −20°C until further use. The thawed samples were loaded into a Fluidigm Biomark 48.48 IFC microfluidic chip (South San Francisco, CA) according to the manufacturer’s instructions. In brief, the samples were diluted to a volume of 45 μl using TE buffer. 2.7 μl of diluted sample was added to 3.3 μl of a master mix consisting of 3.0 μl universal PCR Master Mix and 0.3 μl 20xGE Sample Loading Reagent. Separately, 3 μl of individual primers were mixed with 3 μl 2xGE Assay Loading Reagent. 5 μl of sample, and 5 μl of primer, were loaded into the appropriate well on the Fluidigm chip. The primer list is provided in the Supplementary Table S3. The microfluidic chip was then run on a Biomark real-time PCR instrument, and the raw Ct data were obtained using the Fluidigm software. For data analysis, the qRT-PCR results were normalized against the housekeeping gene B2M to derive ΔC_t_, where ΔC_t_ = C_t B2MG_, and C_t_ represents the number of PCR cycles required to exceed a certain threshold. Expression level is then calculated as Expression = 2^(−ΔCt)^* 1,000. The resulting expression level had a constant of one added to it, and was subsequently converted into Log_2_. When examining the impact of protein hydrogels to cells in culture, Matrigel-methylcellulose medium (MM) was used as the baseline control, whereas colony morphologies used MM ring as the baseline control. Fold change for each individual colony was compared against the baseline using the formula: Log_2_FC = Log_2_(A)—Log_2_(B), or in Microsoft Excel format = log (A, 2)–log (B, 2). For this study, A indicates the expression of a single colony, and B indicates the median expression value of the baseline control. Because of the presence of outliers, the median was used to approximate baseline expression instead of the mean.

*Statistical analysis*. Statistical significance was determined by unpaired, two-tailed Student’s t-test with Welch’s correction between PEP-FN or PEP-LAMA3 *versus* Matrigel-methylcellulose, and budding ring, proto-ring, or grape-like *versus* MM Ring using GraphPad Prism version 9.5.0. *p*-values <0.05 were considered significant. Data are presented as individual datapoints, with a red bar indicating median. Sample group size (n) is indicated in each figure legend.

Additional materials and methods are described in the Supporting Information.

## Results

*Design and expression of PEP-FN and PEP-LAMA3.* We previously reported the creation of a recombinant triblock protein (PEP) capable of forming 3D hydrogels (Shen et al., 2006; Olsen et al., 2010). This protein consists of two leucine zippers (P) derived from rat cartilage oligomeric matrix protein (Efimov et al., 1996) separated by an elastin-like polypeptide (E) consisting of repeating units of the sequence VPGXG, where X can be any amino acid other than proline (Varanko et al., 2020). The P domains form pentameric bundles, leading to physical crosslinking of individual strands and formation of a gel. A characteristic of elastin-like polypeptides is lower critical solution temperature (LCST) behavior, which causes separation of protein-rich phases under conditions of high temperature and high salt, allowing enrichment of these proteins at scale by thermal cycling. Although we have examined carefully the mechanical behavior of hydrogels prepared from PEP and several variants (Shen et al., 2006; Olsen et al., 2010; Dooling and Tirrell, 2016), these proteins have not previously been functionalized with cell-binding motifs or tested in cell culture.

**FIGURE 1:**
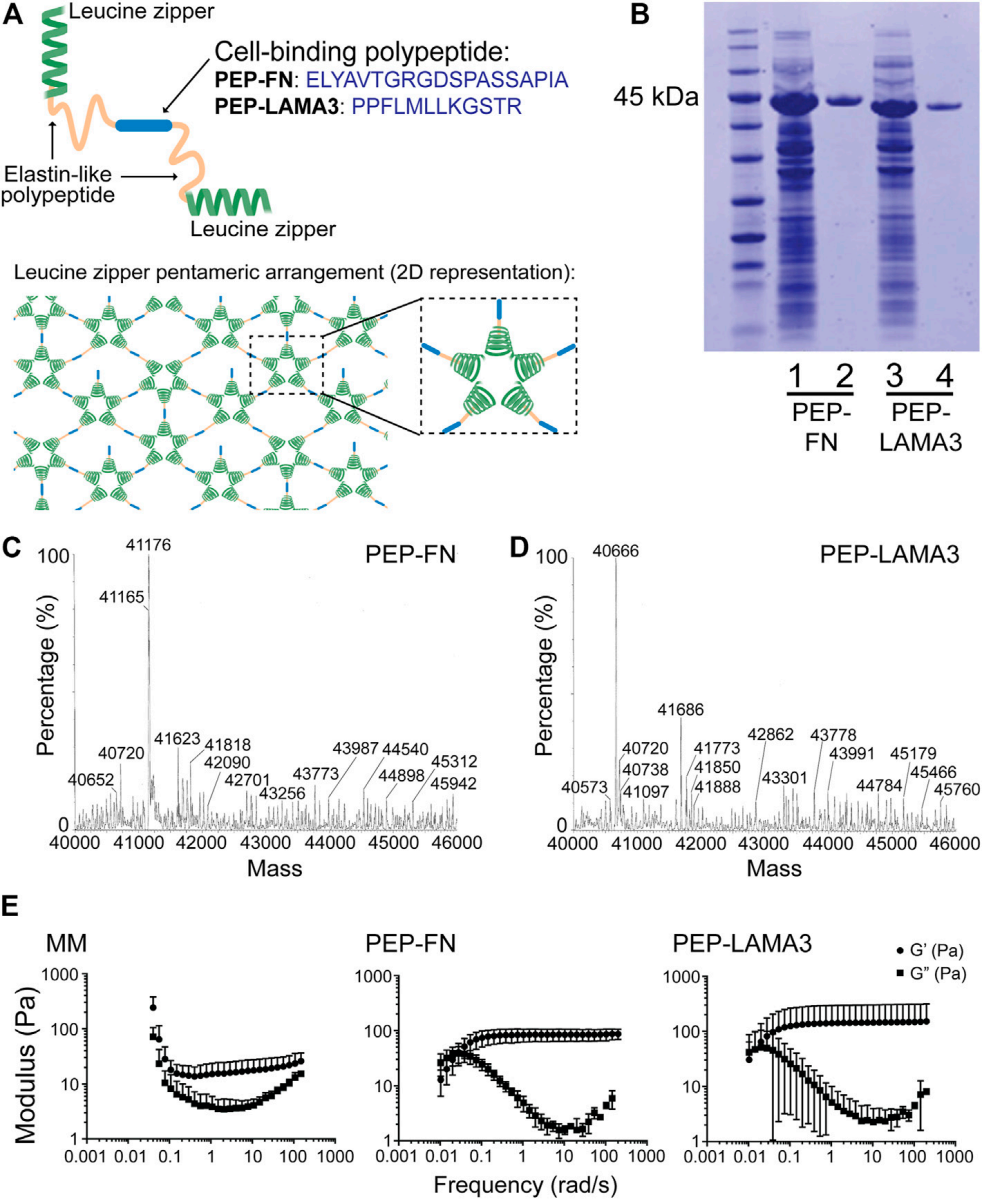
Construction and characterization of PEP-FN and PEP-LAMA3 hydrogels. **(A)** Schematic drawings of PEP-FN and PEP-LAMA3 proteins, which contain the following main elements: 1) leucine zipper (green) from rat cartilage oligomeric matrix protein which forms bundles (pentamers) in solution, leading to a physically cross-linked gel, 2) elastin-like polypeptides (yellow), and 3) the cell binding peptide (blue; either the fibronectin RGD-containing peptide, or laminin three alpha mimetic), which is at the center of the protein. **(B)** SDS-PAGE gel stained with Coomassie InstantBlue on cell lysate prior to purification (lanes 1 and 3) or after purification (lanes 2 and 4). **(C, D)** ESI-TOF of both proteins showing that the protein products are at the correct molecular weights (∼41 kDa) **(E)** Rheological characterization of Matrigel-methylcellulose, PEP-FN, and PEP-LAMA3 media, as used in experimental concentrations.

Both fibronectin (FN) and laminin (LAM), which engage integrin and other receptors on the cell surface, have been shown previously to support pancreatic islet cell survival (Jiang et al., 2002; Kaido et al., 2004; Pinkse et al., 2006; Hadavi et al., 2018; Llacua et al., 2018; Lan et al., 2020). Therefore, we inserted either a RGD-containing sequence derived from FN or a laminin alpha 3 (LAMA3) mimetic sequence between two elastin-like domains in PEP (Figure 1A); the resulting proteins are designated “PEP-FN” and “PEP-LAMA3”, respectively. An added 6x-histidine tag enabled further purification using a Ni-NTA column after the proteins were expressed in *E. coli* and enriched by thermal cycling. The resulting protein preparations yielded single bands in Coomassie blue stained SDS-PAGE gels (Figure 1B). The expected masses for PEP-FN and PEP-LAMA3 were 41,202 and 40,700 Da, respectively, based on their amino acid sequences (Supplementary Table S1); electrospray ionization time-of-flight (ESI-TOF) mass spectrometry confirmed the actual masses to be 41,176 and 40,666 Da, respectively, both within 0.1% of the expected masses (Figures 1C,D).

*Rheological characterization of PEP-FN and PEP-LAMA3 hydrogels.* Physical properties such as matrix stiffness are known to change the differentiation behavior of cells (Engler et al., 2006; Murphy et al., 2014; Lv et al., 2015). We therefore characterized the rheological properties of PEP-FN and PEP-LAMA3 hydrogels (Figure 1E) and compared them to the properties of a medium containing 5% (v/v) Matrigel and 1% (w/v) methylcellulose (referred to as “Matrigel-methylcellulose [MM] medium”), which we previously established for pancreatic cell culture (Jin et al., 2013). We knew from our previous studies that MM medium keeps dissociated single cells from re-aggregating (Jin et al., 2013; Jin et al., 2014), but its rheological properties had not been determined. We found that MM medium displayed an elastic modulus (G’) of approximately 20 Pa across a range of frequencies from 0.1 to 100 rad/s. PEP-FN and PEP-LAMA3 hydrogels were slightly stiffer, with elastic moduli of approximately 80 and 120 Pa, respectively, when measured at a concentration of 2% w/v. All three media allow convenient handling of cells in the procedures used in this work.

*Protein hydrogels prevent cellular aggregation.* To determine whether single cells plated in our protein hydrogels may re-aggregate, we placed dissociated pancreatic cells from young mice in PEP-FN, PEP-LAMA3 or in DMEM-F12 medium that did not contain a gel-forming agent such as PEP-FN, PEP-LAMA3, or Matrigel. Cells were monitored under a wide-field light microscope, and images taken every 10 min over 17 h. We found that in the suspension culture the cells readily aggregated (Supplementary Movie S1), whereas PEP-FN and PEP-LAMA3 hydrogels prevented cell aggregation or movement (Supplementary Movies S2, S3). This result implies that cell clusters or colonies (see below) formed from dissociated pancreatic cells would be the result of growth, rather than re-aggregation, of cells that were seeded.

**FIGURE 2:**
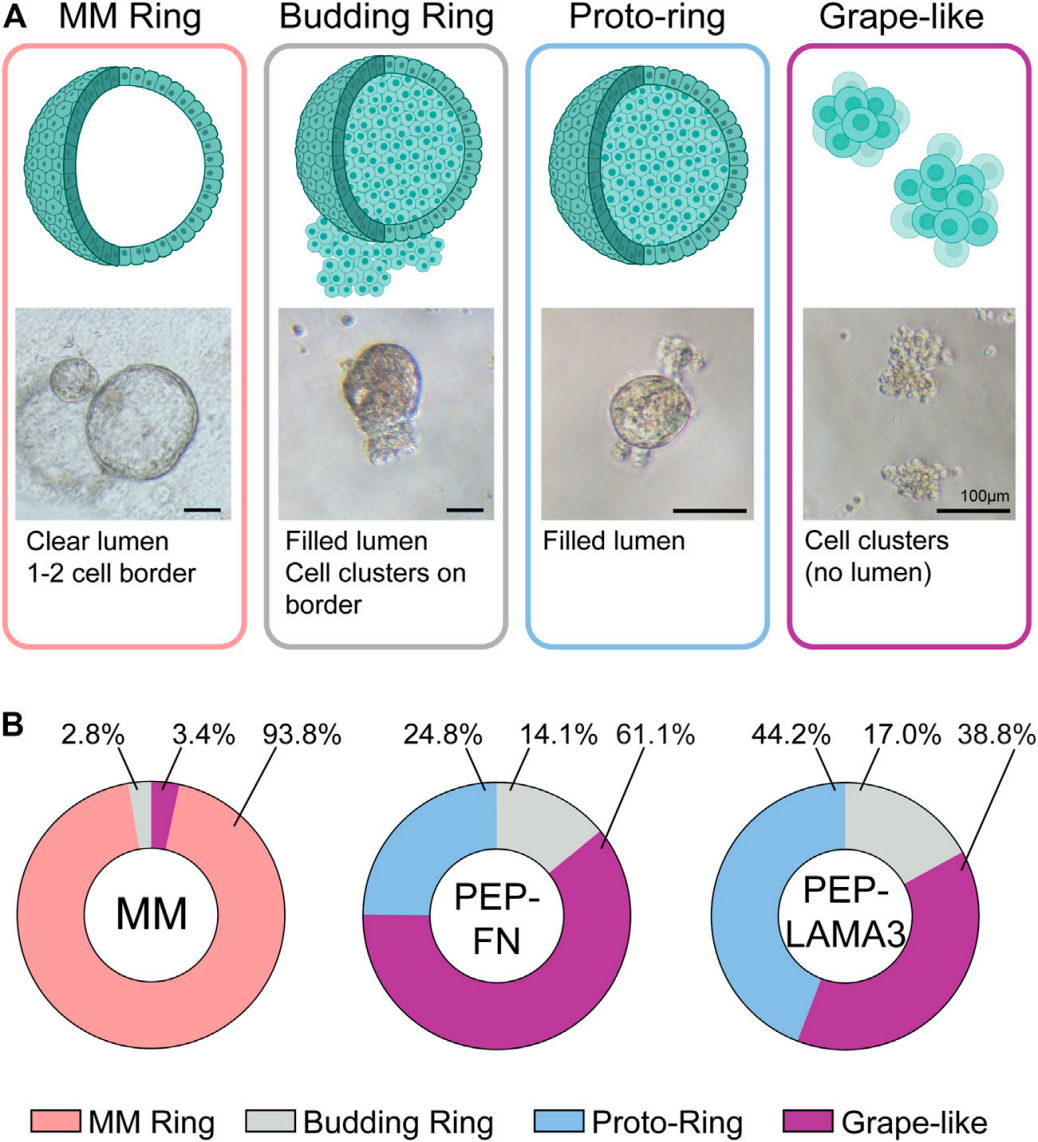
PEP-FN and PEP-LAMA3 hydrogels support the formation of novel types of colonies from pancreatic progenitor cells from 8-day-old mice. **(A)** Representative photomicrographs of various colony types. Cartoon rendition and short description of each type is also shown **(B)** The proportion of each colony type grown in different materials is shown. Supplementary Table S4 (i.e. “Data were derived from a total of three to six replicates from three experiments, see Supplementary Table S4.”

*Novel colony types are found in PEP-FN and PEP-LAMA3 hydrogels.* In our previous studies (Jin et al., 2013), cystic colonies, which we name “MM Ring” here, were the most commonly-observed colonies when pancreatic cells were plated in MM medium. MM Ring colonies consist of large, bright cysts with cells that are not granular at day 4 post-plating. When cells were grown in PEP-FN or PEP-LAMA3, we observed several new types of colonies by phase-contrast microscopy after 7 days in culture (Figure 2A). The new morphologies were designated “grape-like”, “budding ring”, or “proto-ring”. Grape-like colonies are composed of small, barely touching cells. Budding rings are ring colonies that appear to have several cells attached to the ring, and either lack lumen formation, or have a “filled” lumen. Proto-rings are similar to budding rings, but lack external cell attachment. The proto-rings also appear to have internal granules, in contrast to MM Rings, which appear smooth. Distributions of each colony type grown in the three types of media were determined (Figure 2B). The raw data are presented in Supplementary Table S4. As expected, MM medium significantly favored the growth of MM Ring colonies. Interestingly, both PEP-FN and PEP-LAMA3 hydrogels supported the growth of other colony types.

**FIGURE 3:**
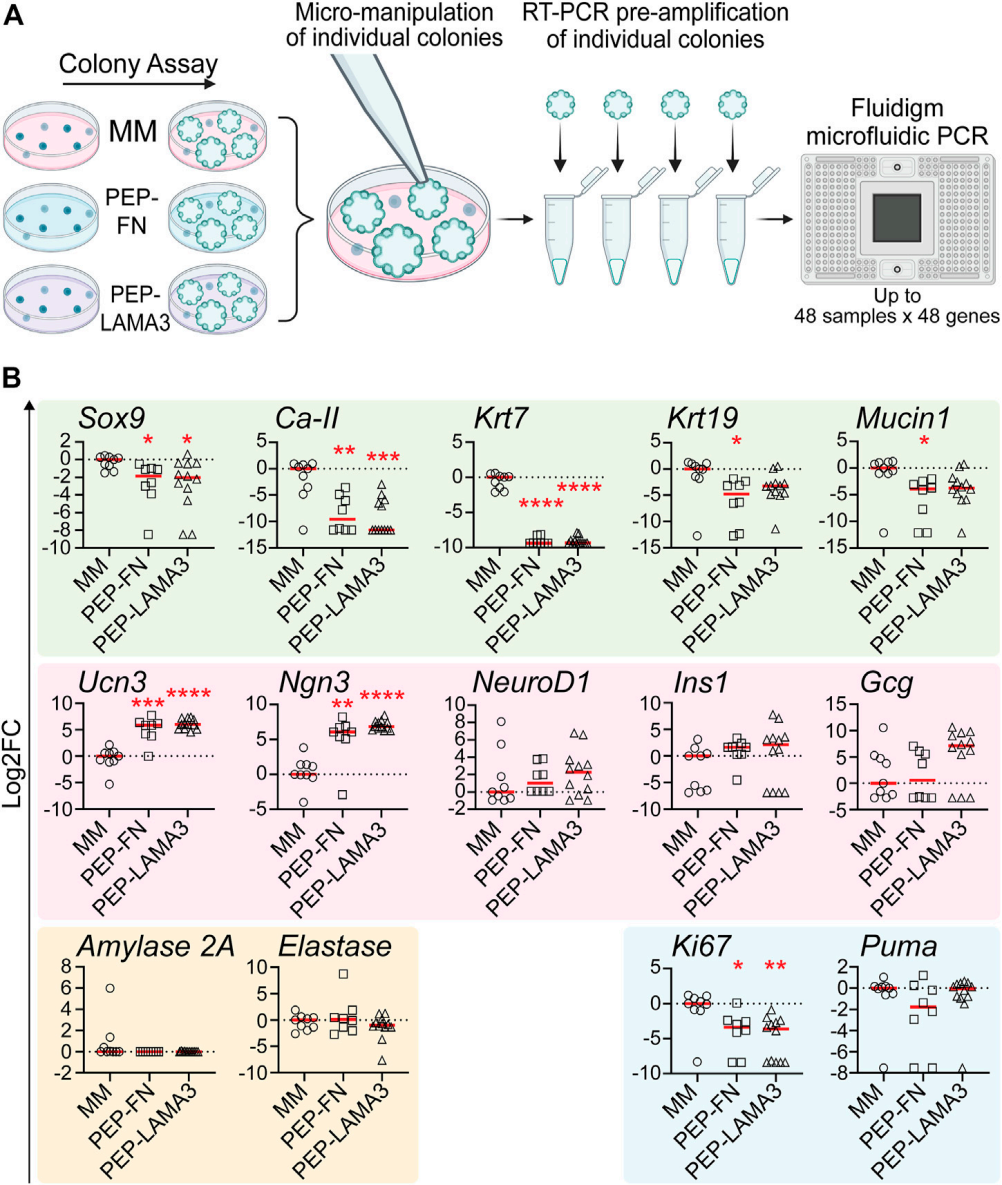
PEP-FN and PEP-LAMA3, compared to Matrigel-methylcellulose medium, support pancreatic cells that express higher levels of endocrine and endocrine progenitor cell markers, and lower levels of ductal cell markers. **(A)** Experimental scheme. Pancreases from 8-day-old mice were dissociated into a single cell suspension and plated into colony assays containing Matrigel-methylcellulose (MM), PEP-FN or PEP-LAMA3. The resulting colonies were visualized under a light microscope and individually lifted one-by-one. mRNA from each colony was converted into cDNA in a single-step RT-PCR reaction. Pre-amplified cDNA was run in a microfluidic PCR chip, where multiple genes expressed by a colony were detected simultaneously **(B)** Gene expression analysis using microfluidic qRT-PCR on individual colonies grown in different cultures. Data were first analyzed relative to the internal control housekeeping gene, B2M. Subsequently, the data were transformed into Log_2_ scale and normalized to the median of the expression levels from colonies grown in the control MM medium. Student’s t-test with Welch’s correction was used to determine the significance of the values between PEP-FN vs. MM and PEP-LAMA3 vs. MM media. Data were derived from a total of 8–12 colonies grown in various media. Each dot represents a colony and the red line represents the median. Red asterisks indicate statistical significance when compared against the control MM culture: *p < 0.05, **p < 0.005, ***p < 0.005, and ****p < 0.0005.

*Gene expression patterns in individual colonies are influenced by the materials in which they grow in culture.* To investigate the influence of culture materials on the gene expression of cells within individual colonies, we performed micromanipulation by observing a colony under a light microscope, then using a micro-pipette with a narrow opening to lift individual colonies and subject them to microfluidic qRT-PCR analysis (Figure 3A), which allows for gene expression analysis from as little as 1 cell (Jin et al., 2013). Compared to those grown in the MM medium, colonies grown in both PEP-FN and PEP-LAMA3 expressed lower levels of ductal markers, *Sox9* (Kopp et al., 2011), carbonic anhydrase-2 (*Ca-II*), and cytokeratin-7 (*Krt7*) (Inada et al., 2006; Pujal et al., 2009). Expression of other ductal markers cytokeratin-19 (*Krt19*) (Qadir et al., 2020) and *Mucin1* (Jonckheere et al., 2010) were also lower in PEP-FN colonies compared to colonies from MM medium (Figure 3B). The endocrine maturation marker, urocortin-3 (*Ucn3*) (Pechhold et al., 2009), and endocrine progenitor cell marker, neurogenin-3 (*Ngn3*) (Gradwohl et al., 2000; Gu et al., 2002), were significantly upregulated by PEP-FN and PEP-LAMA3. Other endocrine markers, neurogenic differentiation 1 (*NeuroD1*), insulin-1 (*Ins1*), and glucagon (*Gcg*) trended higher in colonies supported by PEP-LAMA3 compared to MM, but the difference did not reach significance (Figure 3B). Acinar lineage markers, *Amylase 2a* and *Elastase*, were not changed (Figure 3B). The proliferation marker *Ki67* was downregulated in colonies from both PEP-FN and PEP-LAMA3, compared to MM medium (Figure 3B). The lowered expression of *Ki67* is consistent with prior findings that indicate inhibition of proliferation is required for lineage differentiation of progenitor cells during pancreas development (Hart et al., 2003; Murtaugh et al., 2003; Miyatsuka et al., 2011; Shih et al., 2012). The apoptosis marker *Puma* (Yu and Zhang, 2008) was also not changed in colonies grown in PEP-FN and PEP-LAMA3 compared to MM medium (Figure 3B), suggesting that PEP-FN and PEP-LAMA3 are not toxic to the cells. Together, these results suggest that both PEP-FN and PEP-LAMA3 cultures promote the growth of endocrine lineage cells, whereas MM medium promotes the growth of ductal cells.

**FIGURE 4:**
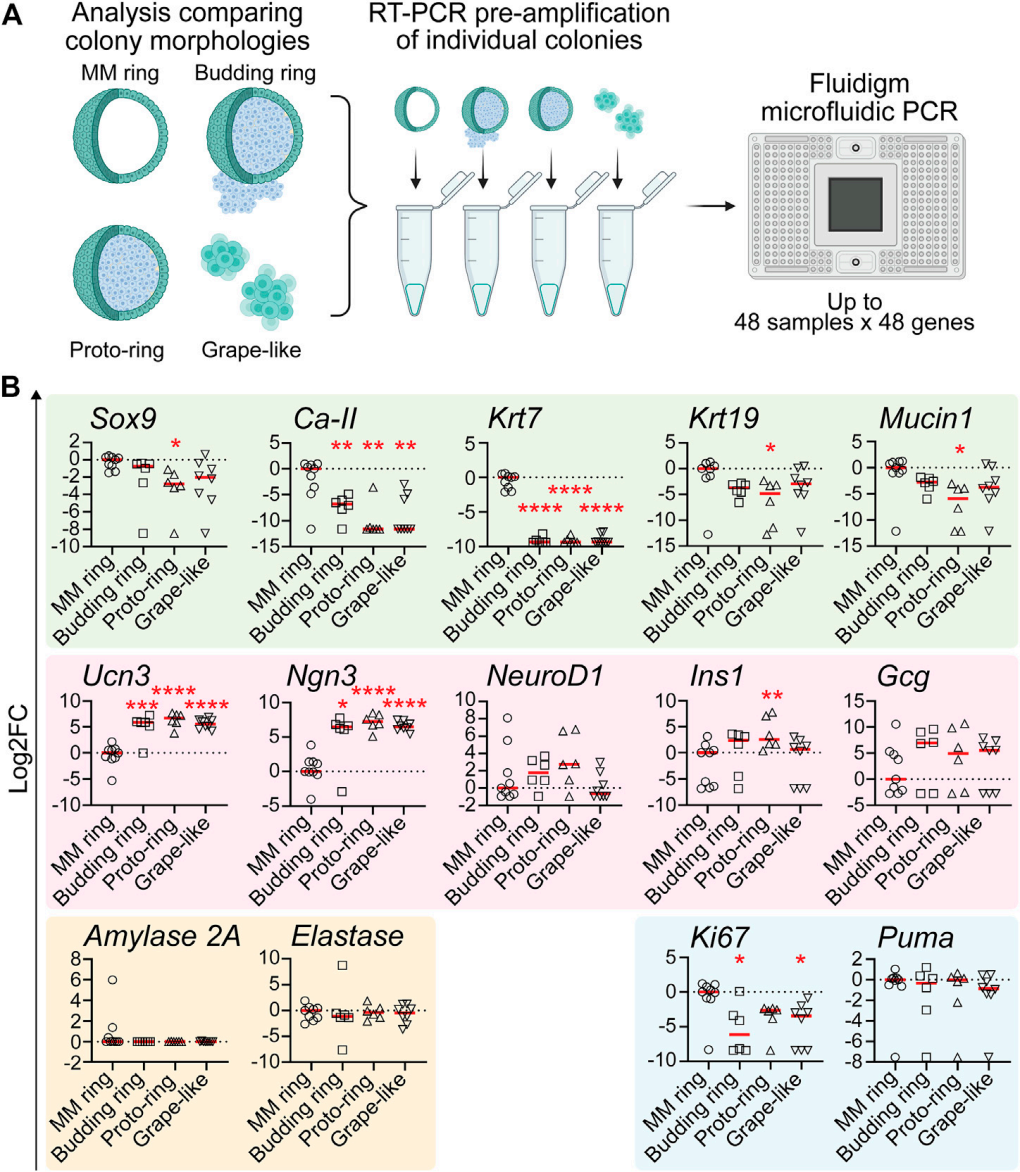
Pancreatic colonies with morphology of “budding ring”, “proto-ring” or “grape-like” express higher levels of endocrine and endocrine progenitor cell markers and lower levels of ductal cell markers **(A)** Experimental scheme. The procedures were the same as explained in Figure 3A, except that the morphology of each colony was also identified and recorded when visualized under a light microscope before performing pre-amplification and microfluidic PCR **(B)** Gene expression analysis using microfluidic qRT-PCR on individual colonies with various morphologies. Data were first analyzed relative to the internal control housekeeping gene, B2M. Subsequently, the data were transformed into Log_2_ scale and normalized to the median expression of the colonies with the MM ring morphology. Student’s t-test with Welch’s correction was used to determine the significance of the values between various morphologies vs. MM ring. Data were derived from a total of six to nine colonies of each morphology. Each dot represents a colony and red line represents the median. Red asterisks indicate statistical significance when compared against the control MM ring morphology: *p < 0.05, **p < 0.005, ***p < 0.005, and ****p < 0.0005.

*qRT-PCR reveals that grape-like, budding ring, and proto-ring colonies are enriched for endocrine lineage markers.* Because PEP-FN and PEP-LAMA3 supported higher proportions of grape-like, budding ring, and proto-ring colonies (Figure 2B), and PEP-FN and PEP-LAMA3 supported cells with higher endocrine gene expression (Figure 3), we predicted that grape-like, budding ring, and proto-ring colonies have higher expression levels of endocrine lineage markers. To test this prediction, we separated the colonies analyzed in Figure 3 based on their morphologies and reanalyzed the data (Figure 4A). MM Ring colonies were used as a comparison because this morphology has been well characterized in our previous studies using MM medium (Wedeken et al., 2017). We found that the ductal markers *Ca-II* and *Krt7* were significantly downregulated in budding ring, proto-ring, and grape-like colonies relative to MM Ring colonies. The ductal markers *Mucin1*, *Krt19*, and *Sox9* were also downregulated in proto-ring colonies compared to MM Ring colonies (Figure 4B). In contrast, the endocrine progenitor marker *Ngn3* and endocrine maturation marker *Ucn3* were upregulated in budding ring, proto-ring, and grape-like colonies, compared to MM Ring colonies. Interestingly, *Ins1* was upregulated in proto-ring colonies, compared to MM Ring colonies (Figure 4B). Compared to MM ring colonies, the proliferation marker *Ki67* was downregulated in budding ring and grape-like, but not proto-ring, colonies (Figure 4B). There was no statistically significant difference observed in the expression of the apoptosis marker *Puma* (Figure 4B). Together, these results suggest that grape-like, budding ring, and proto-ring, colonies are more endocrine in nature, and grape-like and budding ring colonies are post-mitotic compared to MM Ring colonies, which are more duct-like and proliferative.

**FIGURE 5:**
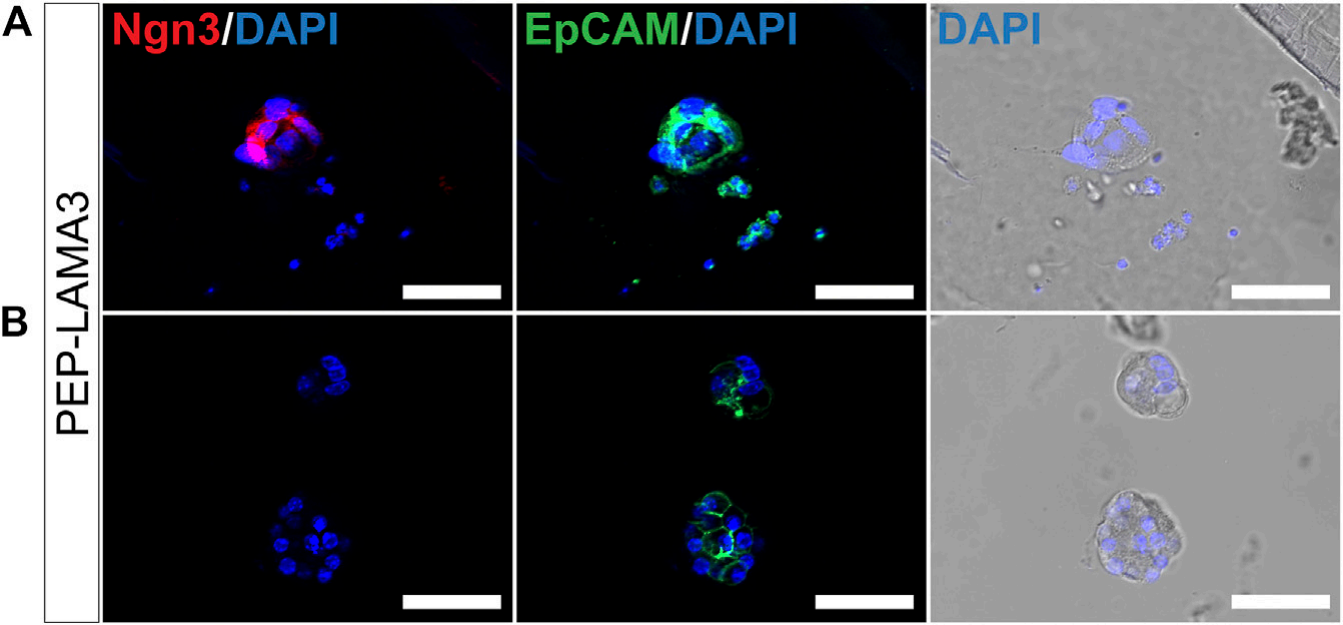
Whole-mount immunofluorescence staining reveals that some cells grown in PEP-LAMA3 express Ngn3, an endocrine progenitor marker. **(A)** Photomicrographs of cells in a colony grown from PEP-LAMA3 expressing the endocrine progenitor marker Ngn3 **(B)** Photomicrographs of other cells grown from PEP-LAMA3 that were handled as in **(A)** but did not express Ngn3. The positive control marker, EpCAM, was used to identify pancreatic epithelial cells. DAPI nuclear staining was overlaid with the brightfield images of the colonies. Scale bars = 50 µm.

*Colonies grown in PEP-LAMA3 express neurogenin3 and chromogranin A proteins.* During development, Ngn3 is expressed transiently in endocrine progenitor cells, with peak expression at embryonic day 15.5 (Gradwohl et al., 2000; Gu et al., 2002; Van de Casteele et al., 2013). A lower level of Ngn3 expression persists into adulthood. We were particularly interested in the presence of Ngn3 in colonies supported by our protein hydrogels indicated by the qRT-PCR (Figure 3). Ngn3-positive cells have previously been difficult to culture, and having a method to obtain such cells may aid in future studies that aim to understand pancreatic endocrine differentiation and maturation. Due to the more significant effect of PEP-LAMA3 compared to PEP-FN on endocrine lineage marker expression (Figure 3B), we focused our attention on colonies grown using PEP-LAMA3. We performed whole-mount immunofluorescence (IF) staining on pooled colonies using antibodies against Ngn3 and observed that some cells from PEP-LAMA3 stained positive for Ngn3 (Figure 5A). No Ngn3 staining was observed in other cells of the same batch, which were handled and washed in the same way (Figure 5B), suggesting that the red/Ngn3 signal observed in Figure 5A is specific.

Pancreatic lineage cells are derived from the definitive endoderm during development and express epithelial cell markers (McCracken and Wells, 2012). To test whether the cells that did not express Ngn3 in Figure 5B were of epithelial cell origin, we co-stained EpCAM, an epithelial cell marker (Trzpis et al., 2007; Keller et al., 2019). Pancreatic cells grown from the PEP-LAMA3 medium expressed EpCAM (Figures 5A,B), suggesting retention of epithelial cell identity.

**FIGURE 6:**
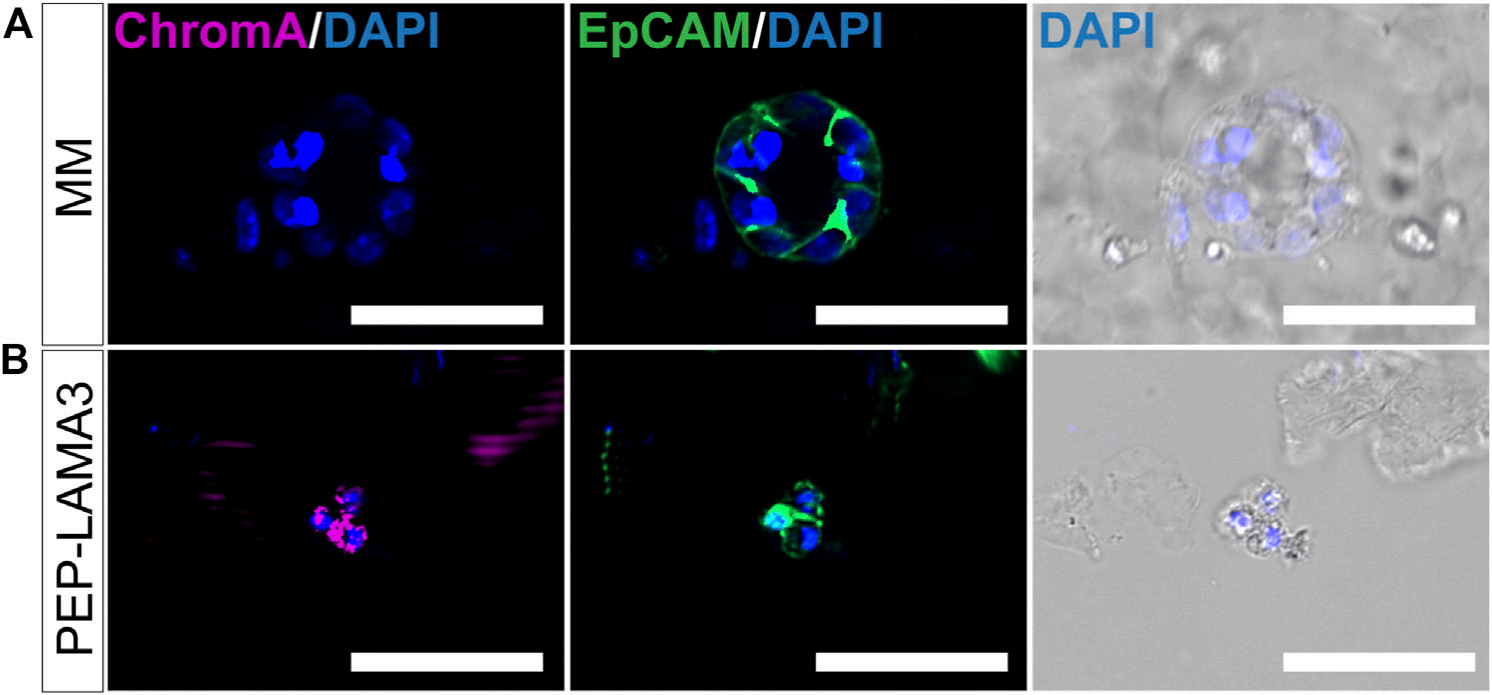
Whole-mount immunofluorescence staining reveals that some cells grown in PEP-LAMA3 express chromogranin A, a pan-endocrine lineage cell marker. **(A)** Photomicrographs of a MM ring colony stained negative for ChromA, a pan-endocrine marker, but positive for the epithelial cell marker, EpCAM **(B)** Photomicrographs of a grape-like colony stained positive for both ChromA and EpCAM. DAPI nuclear staining was overlaid with the brightfield images of the colonies. Scale bars = 50 µm.

Finally, we tested the protein expression of a pan-endocrine marker, chromogranin A (ChromA) (Facer et al., 1985; Deftos, 1991; Portela–Gomes and Stridsberg, 2001; Portela-Gomes et al., 2008), which we also co-stained with EpCAM. As expected, EpCAM was expressed by MM ring colony from MM medium (Figure 6A) and grape-like colony from PEP-LAMA3 (Figure 6B). However, ChromA was only detected in grape-like but not in the MM ring colony. These results confirmed the presence of endocrine-like cells grown in PEP-LAMA3 hydrogel.

## Discussion

In this study, we demonstrate the utility of two novel, well-defined, protein-based hydrogels (i.e., PEP-FN and PEP-LAMA3) in supporting the survival and growth of primary endocrine cells and endocrine progenitor cells in 3D space and in the absence of Matrigel. Although 3D pancreatic organoids have been described using various complex materials, including high concentrations of Matrigel (Huch et al., 2013), decellularized small-intestinal tissues (Giobbe et al., 2019), and decellularized pancreatic tissues (Sackett et al., 2018), our results are consistent with other emerging studies in the stem and progenitor cell field that show that it is possible to grow various progenitor cell types in 3D culture without Matrigel. In particular, synthetic and protein hydrogels can be tuned for their chemical and mechanical properties so that the microenvironment surrounding the cells can be better controlled, manipulated and studied (Kozlowski et al., 2021).

The triblock PEP protein, which may be regarded as the parent of the PEP-FN and PEP-LAMA3 protein hydrogels reported here, has been characterized previously in terms of its mechanical properties and strand exchange dynamics (Olsen et al., 2010; Dooling and Tirrell, 2016; Rapp et al., 2017; Rapp et al., 2018). The material properties of PEP can be modified by introduction of point mutations in the terminal leucine zippers: Dooling and co-workers compared six single-site variants in the P domain, and found I58A to accelerate stress relaxation (Dooling and Tirrell, 2016). In this study, we chose the I58A mutant to match the stiffness of the Matrigel-methylcellulose medium as closely as possible. We found that 2% solutions of PEP-I58A functionalized with FN or LAMA3 domains form semisolid matrices with elastic moduli in the range of 80–120 Pa, comparable to the ideal modulus of approximately 100–300 Pa for human forebrain (Bejoy et al., 2018) and human and mouse intestine (DiMarco et al., 2015; Gjorevski et al., 2016; Cruz-Acuña and Quirós, 2017), and to that of the Matrigel-methylcellulose medium, which is known to support the growth of primary murine pancreatic ductal progenitor cells (Wedeken et al., 2017). Interestingly, the moduli of the materials used here are roughly an order of magnitude lower than that of healthy human pancreas, which is approximately 1,000 Pa (Rubiano et al., 2018). Disease states of the pancreas are associated with stiffer tissues, which can become as high as 5 kPa in tumors (Rubiano et al., 2018). However, these measurements were made on the whole human pancreas, not just the islets of Langerhans or progenitor cells. It may be the case that the islets or the progenitor cell niche and their surrounding ECM are in fact softer than the pancreas as a whole. Future experiments using a gradient of elastic moduli will likely find that cell fate is dependent on matrix stiffness, and proteins of the PEP type with different point mutations in the leucine zipper may be instrumental in understanding these effects.

Human pluripotent stem cell derived pancreatic progenitor (PP) cells and beta like cells (SC-β cells) (Melton, 2021) as well as adult human and rodent islets have been used to model endocrine differentiation and maturation *in vitro* (Jiang et al., 2022). Although human NGN3+ endocrine progenitor cells can be differentiated from stem cells without exogenous ECM proteins, the subsequently differentiated beta-like cells are still immature in functionality compared to adult human beta cells (Hrvatin et al., 2014; Maxwell et al., 2022). In fact, gene expression profiling on SC-β cells have revealed a gene signature that resembles human fetal beta cells (Hrvatin et al., 2014), which are immature. On the other hand, studies that use primary adult islet cells cannot address the question of functional maturity because these adult cells are already terminally differentiated and matured. The maturation process of beta cells is not initiated until after birth (Blum et al., 2012), when postnatal development is marked by changes in diet, nutrient metabolism and the hormonal milieu (Wortham and Sander, 2021). Accordingly, murine beta cells become functionally mature between day 1 and day 15 after birth (Blum et al., 2012). Therefore, in this work we chose murine postnatal day 8 pancreatic cells to identify biomaterials capable of supporting the survival of the primary Ngn3-expressing endocrine progenitor cells and endocrine cells in a 3D culture platform.

We find that murine postnatal pancreatic cells arising from culture in both PEP-FN and PEP-LAMA3 hydrogels are endocrine in nature, in contrast to cells grown in Matrigel, which favors the ductal cell type. Previously, we functionalized an elastin-like polypeptide (ELP) with an IKVAV-containing sequence derived from laminin alpha 1, which we designated artificial (a)ECM-lam. Because aECM-lam alone did not form a hydrogel, we mixed it at a final concentration of 100 ug/mL in 1% (w/v) methylcellulose for cell culture. Using this culture system, we showed that aECM-lam, but not the control ELP fitted with a scrambled IKVAV sequence, favored the formation of not only endocrine but also acinar cells from pancreatic progenitor cells (Jin et al., 2013; Ghazalli et al., 2015). This result, together with those reported here, highlights the importance of cell-binding domains in cell fate determination, as demonstrated in other progenitor cell types (Leipzig et al., 2011; Brown and Badylak, 2014; Jann et al., 2020). However, whether a PEP functionalized with the laminin alpha one IKVAV sequence may also support the dual fates of endocrine and acinar cells awaits further investigation.

There are several limitations with our current studies. First, we determined that the presence of FN and LAMA3 motifs were sufficient for formation of the endocrine colonies, but we did not show that they were necessary, by using a PEP construct without the presence of a signaling sequence or scrambled sequences. A prior study has shown that, without functionalization, a PEG-based gel crosslinked with amikacin, known as Amikagel, can support stem cell derived PP cells reaggregation and spontaneous differentiation (Candiello et al., 2018). Second, the lessons we have derived from this study are limited to young mice: we did not test using either murine embryonic, or human stem cells. Finally, we did not study mechanical effects on differentiation. Considering that the COMPcc coiled-coil’s binding affinity is tunable (Dooling and Tirrell, 2016; Rapp et al., 2017), the mechanical properties of both PEP constructs could be altered without changing the chemical properties of the culture medium. Considering recent advances connecting stem cell differentiation to changes in mechanical properties (Han et al., 2020; Hayward et al., 2021; Zhang et al., 2022), this may be a fruitful area of further research.

In summary, our study provides a proof-of-concept cell culture matrix for endocrine cell differentiation in young mice without Matrigel. This work sets the stage/enables the further development of hydrogel matrices for other cell models with tunable physical properties for optimization. We have shown that primary endocrine and endocrine progenitor cells isolated from day-8 murine pancreas can be grown in PEP-FN and PEP-LAMA3 recombinant protein hydrogels in 3D space as colonies. Our well-defined protein hydrogel system will be an ideal platform to further define signaling required for maturation of pancreatic beta cells needed for replacement therapy of T1D.

## Data Availability

The original contributions presented in the study are included in the article/Supplementary Material, further inquiries can be directed to the corresponding authors.
