# Supplementary material for: A matrigel-free method for culture of pancreatic endocrine-like cells in defined protein-based hydrogels
Source: Front Bioeng Biotechnol. 2023 Mar 9;11:1144209. doi: 10.3389/fbioe.2023.1144209 (PMC10033864; doi:10.3389/fbioe.2023.1144209)
Supplement: Supplementary file 4 [file DataSheet1.DOCX]

Supporting Information for “**A Matrigel-free Method for Culture of Pancreatic Endocrine-like Cells in Defined Protein-based Hydrogels”**

Mark T. Kozlowski, Heather N. Zook, Desnor N. Chigumba, Christopher P. Johnstone, Luis F. Caldera, Hung-Ping Shih, David A. Tirrell, Hsun Teresa Ku

This file contains the following information:

1. Supplementary materials and methods.
2. Supplementary figure 1.
3. Supplementary tables 1-4.
4. Supplementary movie legends.
5. References cited.

**SUPPLMENTARY MATERIALS AND METHODS**

***Plasmid construction.***

DNA constructs encoding both proteins were cloned in *E. coli* strain DH10B, using recursive ligation into a pQE80-L plasmid (Qiagen, Hilden, Germany) (**Supplementary Figure 1**). In short, the leucine zipper P-containing vector “pLJD1” on a pQE80-L backbone was digested with XhoI and HindIII (New England Biolabs, Ipswich, MA). The “pLJD2” vector containing the elastin-like polypeptide E6 ([(VPGAG)_2_VPGEG(VPGAG)_2_]_6_) was digested using SalI and HindIII. As SalI and XhoI leave complementary sticky ends, ligation of the resulting inserts from the two vectors generated a new vector, pMTK-PE. This vector was subsequently digested with XhoI and HindIII, allowing for the insertion of DNA fragments encoding the fibronectin or laminin alpha 3 peptide sequences. The full-length sequences of PEP-FN and PEP-LAMA3 are presented in **Supplementary Table 1**. The LAMA3 peptide was previously reported by Tjin and co-workers ([Tjin et al., 2014](#_ENREF_2)). Plasmids were electroporated into *E. coli* strain DH10B and bacterial clones were selected on LB medium plates supplemented with 100 mg/L carbenicillin. All antibiotics were obtained from BioPioneer Inc. (San Diego, CA), dissolved at 1000X working concentration, and sterile filtered.

To produce plasmids for sequencing, bacterial colonies were propagated overnight in LB medium supplemented with 100 mg/L carbenicillin, and plasmids were isolated using a Qiagen QiaSpin DNA miniprep kit according to manufacturer’s instructions. The clone sequences were confirmed by Sanger DNA sequencing (Laragen Inc., Culver City, CA).

***Protein expression and purification.***

To produce large amounts of proteins in quantities sufficient for use in cell culture, verified plasmids were first transformed into *E. coli* strain BL21 by electroporation, and selected for using LB plates supplemented with carbenicillin (100 mg/L). Individual colonies were grown to stationary phase in LB medium supplemented with ampicillin (100 mg/L) by overnight incubation at 37° C. This starter culture was used to inoculate Terrific Broth (TB) medium likewise supplemented with ampicillin, at an inoculation ratio of 50:1. The culture was then incubated at 37°C until it reached an optical density (OD) of 0.6-0.8. Expression of the protein was under the control of the T5 promoter native to pQE80, and was induced by the addition of isopropyl β-D-1-thiogalactopyranoside (IPTG) to a final working concentration of 1 mM. Expression was continued for 4 h and then cells were pelleted by centrifugation at 8,000 g for 8 min. The pellet was resuspended in Buffer B (8 M urea, 100 mM NaH_2_PO_4_, 10 mM Tris base, 10 mM imidazole, adjusted to pH 8 with NaOH) at a ratio of 4 mL of buffer per 1 g of wet bacterial pellet. This suspension was then frozen at -80°C overnight, thawed, and the bacteria were lysed by sonication in a Q500 probe sonicator (Qsonica, Newtown, CT). The bacterial lysate was clarified by centrifugation at 50,000 g for 1 h. The resulting clarified supernatant was adjusted to pH 8.0 by the addition of 6 M sodium hydroxide, and nickel-nitrilotriacetic acid (Ni-NTA, Fisher Scientific, Tustin, CA) resin was added at a ratio of approximately 1 mL of settled resin to 10 mL of clarified supernatant. The mixture was stirred at room temperature for 1 h, then loaded into a chromatography column. The Ni-NTA resin was washed first with 10 column-volumes of cold Buffer B, then with 50 column volumes of cold Buffer B supplemented with 0.1% Triton X-144 (Sigma-Aldrich). Next, the resin was washed with 10 column volumes of Buffer C (10 mM Tris base, 10 mM imidazole, pH 8), followed by alternating 10 column volume washes of 60% isopropanol/Buffer C and Buffer C. This alternation between washes of 60% isopropanol/Buffer C and Buffer C was conducted 3 times. After a final wash with Buffer C, the resin was washed with 10 column volumes of Buffer D (8 M urea, 100 mM Tris, pH 8). The resin was then eluted using 3 column volumes of Buffer D supplemented with 250 mM imidazole. To check enrichment, the flow-through fraction and elution fractions of both proteins were run on a NuPAGE 4-12% Bis-Tris protein gel (ThermoFisher) after denaturation in SDS buffer, and stained with Coomassie InstantBlue (Expedeon, San Diego, CA).

For refolding, the elution fraction was dialyzed against deionized water at 4°C for 48 h at a ratio of approximately 50 parts water to 1 part elution fraction using a dialysis membrane with a molecular weight cutoff of 12-14 kDa (Spectrum Laboratories, Rancho Dominguez, CA). The water was changed three times each day. After dialysis, the elution fraction was sterile filtered in a tissue culture hood through a 0.2 μm syringe filter into a sterile conical tube. The conical tube was then flash-frozen in liquid nitrogen, and lyophilized for 2-3 days. The yield of protein obtained by this method was approximately 100 mg/L of rich medium.

***Endotoxin determination.***

The amount of endotoxin present in the sample was tested using an Invivogen HEK-Blue Endotoxin Standard kit (Invivogen, San Diego, CA), and found to be between 3-10 endotoxin units/mg among preparations.

***Molecular weight determination.***

The molecular weight of the proteins was confirmed using electrospray ionization-time of flight (ESI-TOF) mass spectrometry on an LCT Premier XE electrospray TOF mass spectrometer (Waters, Milford, MA).

**Supplementary Figure 1. Cloning strategy for obtaining PEP-FN and PEP-LAMA3 plasmids.**


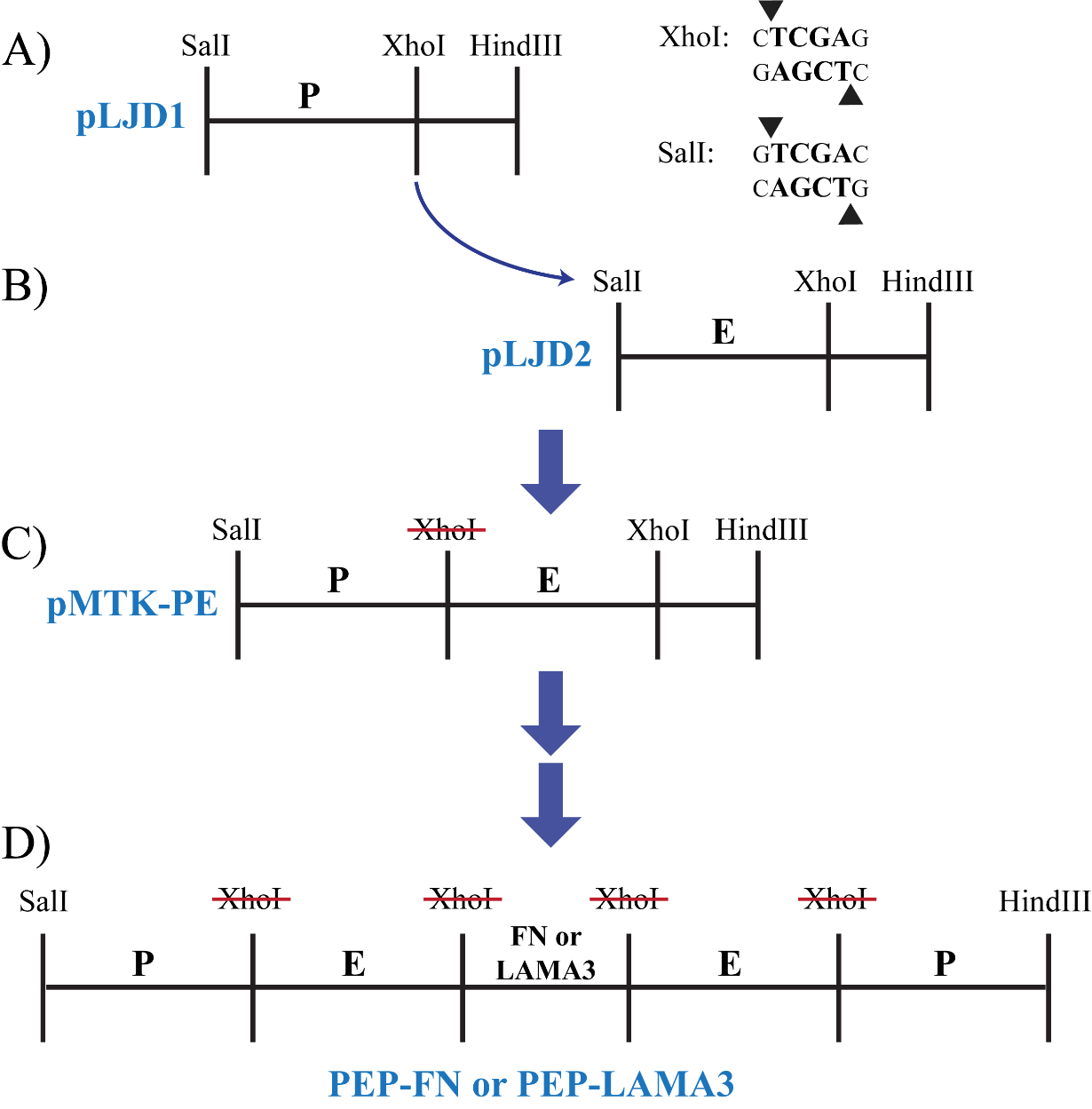


Our cloning strategy uses the fact that the sticky ends of SalI and XhoI are complementary, and that both sites are destroyed when these complementary ends are ligated together. (A) The vector pLJD1 on a pQE80L backbone, which contains a fragment encoding the coil sequence “P”, is digested with XhoI and HindIII. (B) The vector pLJD2, which contains a fragment encoding the elastin-like polypeptide insert “E”, is digested with SalI and HindIII. (C) The sticky ends were then ligated together, resulting in the vector pMTK-PE, and the destruction of the previous XhoI site C-terminal to P. (D) Through three more sequential digestions and ligations, and the insertion of the FN or LAMA3 sequence, the entire construct was made, with destroyed restriction sites marked with red strikeouts.

**Supplementary Table 1. Amino acid sequences for recombinant proteins generated in this study.**

| Protein | Amino Acid Sequence | Molecular Weight |
| --- | --- | --- |
| PEP-FN | MRGSSHHHHHHENLYFQGDLAPQMLRELQETNAALQDVRELLRQQVKEATFLKNTVMESDASGGGSVDVPGAGVPGAGVPGEGVPGAGVPGAGVPGAGVPGAGVPGEGVPGAGVPGAGVPGAGVPGAGVPGEGVPGAGVPGAGLDVPGAGVPGAGVPGEGVPGAGVPGAGVPGAGVPGAGVPGEGVPGAGVPGAGVPGAGVPGAGVPGEGVPGAGVPGAGLDDRP**ELYAVTGRGDSPASSAPIA**TSLDVPGAGVPGAGVPGEGVPGAGVPGAGVPGAGVPGAGVPGEGVPGAGVPGAGVPGAGVPGAGVPGEGVPGAGVPGAGLDVPGAGVPGAGVPGEGVPGAGVPGAGVPGAGVPGAGVPGEGVPGAGVPGAGVPGAGVPGAGVPGEGVPGAGVPGAGLDENLYFQGDLAPQMLRELQETNAALQDVRELLRQQVKEATFLKNTVMESDASGGKLAS | 41,202 Da |
| PEP-LAMA3 | MRGSSHHHHHHENLYFQGDLAPQMLRELQETNAALQDVRELLRQQVKEATFLKNTVMESDASGGGSVDVPGAGVPGAGVPGEGVPGAGVPGAGVPGAGVPGAGVPGEGVPGAGVPGAGVPGAGVPGAGVPGEGVPGAGVPGAGLDVPGAGVPGAGVPGEGVPGAGVPGAGVPGAGVPGAGVPGEGVPGAGVPGAGVPGAGVPGAGVPGEGVPGAGVPGAGLDDRP**PPFLMLLKGSTR**TSLDVPGAGVPGAGVPGEGVPGAGVPGAGVPGAGVPGAGVPGEGVPGAGVPGAGVPGAGVPGAGVPGEGVPGAGVPGAGLDVPGAGVPGAGVPGEGVPGAGVPGAGVPGAGVPGAGVPGEGVPGAGVPGAGVPGAGVPGAGVPGEGVPGAGVPGAGLDENLYFQGDLAPQMLRELQETNAALQDVRELLRQQVKEATFLKNTVMESDASGGKLAS | 40,700 Da |

Shown are the full sequences and molecular weights of the proteins used in this study, referred to as PEP-FN and PEP-LAMA3. Each protein starts with a 6x-histidine tag and contains two leucine zippers derived from rat collagen oligomeric matrix protein and two elastin-like polypeptide sequences, which flank a signaling peptide derived either from fibronectin (in the PEP-FN case) or from laminin 3 alpha (in the PEP-LAMA3 case). The cell-binding peptide sequence is highlighted in red. See Fig. 1A for schematic drawings.

**Supplementary Table 2. List of antibodies used for whole-mount immunostaining.**

| **Antigen** | **Species, conjugation** | **Source** | **Product No.** | **Dilution** | **RRID** |
| --- | --- | --- | --- | --- | --- |
| ***Primary antibodies*** | |  |  |  |  |
| Ngn3 | Guinea pig | Maike Sander, UCSD | N/A | 1:500 | N/A([Henseleit et al., 2005](#_ENREF_1)) |
| Chromogranin A | Goat | Santa Cruz Bio, Santa Cruz, CA | sc-1488 | 1:500 | AB_2276319 |
| EpCAM | Rat | Development Studies Hybridoma Bank (DSHB) | G8.8 | 1:100 | AB_2098655 |
|  |  |  |  |  |  |
| ***Secondary antibodies*** | |  |  |  |  |
| Guinea pig | Donkey, Cy3 | Jackson ImmunoResearch | 706-166-148 | 1:2000 | AB_2340461 |
| Goat | Donkey, AlexFluor 647 | Jackson ImmunoResearch | 705-605-147 | 1:500 | AB_2340437 |
| Rat | Donkey, AlexFluor 488 | Jackson ImmunoResearch | 712-545-153 | 1:1000 | AB_2340684 |

**Supplementary Table 3. TaqMan probes (Thermo Fisher) used for single-colony Fluidigm qRT-PCR analysis.**

| **Primer**  **number** | **Function** | **Gene** | **Cat#** |
| --- | --- | --- | --- |
| 1 | Housekeeping | B2-MG | Mm 00437762_m1 |
| 2 | Acinar | Amylase 2A | Mm02342487_g1 |
| 3 |  | Elastase | Mm00712898_m1 |
| 4 | Duct | Carbonic Anhydrase 2 (Ca-II) | Mm00501572_m1 |
| 5 |  | Cytokeratin 19 (Krt19) | Mm00492980_m1 |
| 6 |  | Cytokeratin 7 (Krt7) | Mm00466676_m1 |
| 7 |  | Mucin1 | Mm00449604_m1 |
| 8 |  | Sox9 | Mm 00448840_m1 |
| 9 | Endocrine progenitor | NeuroD1 | Mm0180117_m1 |
| 10 |  | Neurogenin 3 (Ngn3) | Mm00437606_s1 |
| 11 | Endocrine | Insulin 1 (Ins1) | Mm01259683_g1 |
| 12 |  | Glucagon (Gcg) | Mm00801712_m1 |
| 13 |  | Urocortin 3 (Ucn3) | Mm00453206_s1 |
| 14 | Proliferation | Ki67 | Mm01278617_m1 |
| 15 | Apoptosis | Puma (Bbc3) | Mm00519268_m1 |

**Supplementary Table 4. Raw colony count data.**

| **Condition** | **Experiment** | **Replicate**  **wells** | **MM Ring** | **Budding Ring** | **Proto-Ring** | **Grape-like** | **Total** |
| --- | --- | --- | --- | --- | --- | --- | --- |
| **MM**  (2,500 cells/well) | 1 | 1 | 101 | 6 | 0 | 6 | 113 |
|  |  | 2 | 142 | 9 | 0 | 6 | 157 |
|  |  | 3 | 114 | 4 | 0 | 11 | 129 |
|  | 2 | 1 | 170 | 3 | 0 | 0 | 173 |
|  |  | 2 | 135 | 1 | 0 | 4 | 140 |
|  |  | 3 | 147 | 0 | 0 | 0 | 147 |
| **PEP-FN**  (10,000 cells/well) | 1 | 1 | 0 | 2 | 15 | 34 | 51 |
|  |  | 2 | 0 | 3 | 20 | 39 | 62 |
|  |  | 3 | 0 | 9 | 15 | 48 | 72 |
|  | 2 | 1 | 0 | 1 | 13 | 29 | 43 |
|  |  | 2 | 0 | 3 | 18 | 29 | 50 |
|  |  | 3 | 0 | 0 | 14 | 24 | 38 |
|  | 3 | 1 | 0 | 23 | 25 | 59 | 107 |
|  |  | 2 | 0 | 16 | 11 | 43 | 70 |
|  |  | 3 | 0 | 14 | 15 | 31 | 60 |
|  |  | 4 | 0 | 13 | 14 | 49 | 76 |
|  |  | 5 | 0 | 11 | 8 | 30 | 49 |
|  |  | 6 | 0 | 14 | 6 | 24 | 44 |
| **PEP-LAMA3**  (10,000 cells/well) | 1 | 1 | 0 | 18 | 50 | 47 | 115 |
|  |  | 2 | 0 | 29 | 39 | 21 | 89 |
|  |  | 3 | 0 | 17 | 27 | 29 | 73 |
|  | 2 | 1 | 0 | 13 | 54 | 57 | 124 |
|  |  | 2 | 0 | 15 | 56 | 32 | 103 |
|  |  | 3 | 0 | 6 | 57 | 39 | 102 |
|  | 3 | 1 | 0 | 9 | 23 | 20 | 52 |
|  |  | 2 | 0 | 11 | 30 | 35 | 76 |
|  |  | 3 | 0 | 17 | 45 | 37 | 99 |
|  |  | 4 | 0 | 17 | 35 | 37 | 89 |
|  |  | 5 | 0 | 8 | 19 | 21 | 48 |

Pancreases from 8-day-old B6 mice were dissociated into single cells and plated into PEP-LAMA3 (10,000 cells per well), PEP-FN (10,000 cells per well), or Matrigel-methylcellulose media (2,500 cells per well). The resulting colonies were counted 4 days after plating when grown in Matrigel-methylcellulose medium, while the colonies grown in PEP-LAMA3 and PEP-FN were counted 7 days after plating, and according to their morphologies. Data from a total of 3 independent experiments are shown. Within each experiment, 3-6 technical replicates were used.

**Supplementary Movie Legends**

**Supplementary Movie 1. Dissociated pancreatic cells readily reaggregate in suspension culture.**

A timelapse is shown of cells grown in DMEM-F12, EGF and nicotinamide, without the presence of any gelation agents such as Matrigel, methylcellulose or protein hydrogels. Micrographs were taken once every 10 minutes for over 17 hours, and the film is played back at a rate of 5 frames per second. Note the formation of cellular aggregates over time.

**Supplementary Movie 2. PEP-LAMA3 prevents the reaggregation of dissociated pancreatic cells.**

A 17-hour timelapse is shown of cells grown in PEP-LAMA3. Micrographs were taken once every 10 minutes for over 17 hours, and the film is played back at a rate of 5 frames per second. Note that no reaggregation is observed.

**Supplementary Movie 3. PEP-FN prevents the reaggregation of dissociated pancreatic cells.**

A 17-hour timelapse is shown of cells grown in PEP-FN. Micrographs were taken once every 10 minutes for over 17 hours, and the film is played back at a rate of 5 frames per second. Note that no reaggregation is observed.

**References**

Henseleit, K.D., Nelson, S.B., Kuhlbrodt, K., Hennings, J.C., Ericson, J., and Sander, M. (2005). NKX6 transcription factor activity is required for alpha- and beta-cell development in the pancreas. *Development* 132(13)**,** 3139-3149. doi: 10.1242/dev.01875.

Tjin, M.S., Chua, A.W.C., Ma, D.R., Lee, S.T., and Fong, E. (2014). Human Epidermal Keratinocyte Cell Response on Integrin-Specific Artificial Extracellular Matrix Proteins. *Macromolecular Bioscience* 14(8)**,** 1125-1134. doi: 10.1002/mabi.201400015.
